# An Examination of Recording Accuracy and Precision From Eye Tracking Data From Toddlerhood to Adulthood

**DOI:** 10.3389/fpsyg.2018.00803

**Published:** 2018-05-23

**Authors:** Kirsten A. Dalrymple, Marie D. Manner, Katherine A. Harmelink, Elayne P. Teska, Jed T. Elison

**Affiliations:** ^1^Institute of Child Development, College of Education and Human Development, University of Minnesota, Minneapolis, MN, United States; ^2^Department of Computer Science & Engineering, College of Science & Engineering, University of Minnesota, Minneapolis, MN, United States; ^3^Department of School Counseling, School of Education, North Dakota State University, Fargo, ND, United States; ^4^Department of Comparative Human Development, University of Chicago, Chicago, IL, United States; ^5^Department of Pediatrics, University of Minnesota Medical School, University of Minnesota, Minneapolis, MN, United States

**Keywords:** eye tracking, calibration, development, methods, quality assessment, toddlers

## Abstract

The quantitative assessment of eye tracking data quality is critical for ensuring accuracy and precision of gaze position measurements. However, researchers often report the eye tracker’s optimal manufacturer’s specifications rather than empirical data about the accuracy and precision of the eye tracking data being presented. Indeed, a recent report indicates that less than half of eye tracking researchers surveyed take the eye tracker’s accuracy into account when determining areas of interest for analysis, an oversight that could impact the validity of reported results and conclusions. Accordingly, we designed a calibration verification protocol to augment independent quality assessment of eye tracking data and examined whether accuracy and precision varied between three age groups of participants. We also examined the degree to which our externally quantified quality assurance metrics aligned with those reported by the manufacturer. We collected data in standard laboratory conditions to demonstrate our method, to illustrate how data quality can vary with participant age, and to give a simple example of the degree to which data quality can differ from manufacturer reported values. In the sample data we collected, accuracy for adults was within the range advertised by the manufacturer, but for school-aged children, accuracy and precision measures were outside this range. Data from toddlers were less accurate and less precise than data from adults. Based on an *a priori* inclusion criterion, we determined that we could exclude approximately 20% of toddler participants for poor calibration quality quantified using our calibration assessment protocol. We recommend implementing and reporting quality assessment protocols for any eye tracking tasks with participants of any age or developmental ability. We conclude with general observations about our data, recommendations for what factors to consider when establishing data inclusion criteria, and suggestions for stimulus design that can help accommodate variability in calibration. The methods outlined here may be particularly useful for developmental psychologists who use eye tracking as a tool, but who are not experts in eye tracking *per se*. The calibration verification stimuli and data processing scripts that we developed, along with step-by-step instructions, are freely available for other researchers.

## Introduction

Eye tracking has a long and intriguing history in psychological research (see Karatekin, 2007 for a review). It has become an important tool for evaluating a wide variety of behaviors and cognitive processes, such as information processing speed, attentional orienting, face processing, reading, and aspects of memory, across a variety of populations and ages. In infant research, looking time has been a key measure of perception, cognition, language, and social development (Aslin, 2007). Eye tracking represents a major improvement in spatial and temporal resolution over traditional video-based hand coding of infant looking behavior (c.f., Aslin, 2007 re: the microarchitecture of looking time), and allows for the efficient collection of large quantities of data. Despite these improvements, eye tracking measures remain inexact, and eye tracking quality varies by software/hardware/manufacturer and by the population being studied (e.g., race, Blignaut and Wium, 2014; age, Wass et al., 2014). Variability in data quality can also be exacerbated in special populations, such as infants and young children (Wass et al., 2014), who cannot understand instructions and who may introduce more head and body movements than adults. Yet some developmental researchers may not be experts in eye tracking *per se* and may instead use it as a tool to answer questions about development, not appreciating the importance of eye tracking data quality and/or how to measure it.

The issue of eye tracking data quality is particularly important because systematic differences in data quality can influence key dependent measures that, if not carefully assessed, can be misinterpreted as differences between participant groups or experimental conditions (Wass et al., 2014). Researchers have recently begun assessing various parameters that affect the quality of infant eye tracking data. For example, Niehorster et al. (2017) evaluated the performance of a variety of remote eye tracking systems while tracking unrestrained participants. They found that these eye trackers can suffer data loss and other issues that can lead to errors in data analysis, even when the participant is within the recommended tracking area. They recommend in-house assessments for researchers to evaluate their equipment. Hessels et al. (2015) evaluated the effect of infant eye color, positioning, and head movement on data quality and offered useful tips for improving data acquisition. While helpful for reducing error, even with ideal experimental settings, some measurement error still occurs. This has led others to advocate for standard reporting of data quality and the implementation of quality assessment measures (Oakes, 2010; Blignaut and Wium, 2014; Wass et al., 2014).

**FIGURE 1:**
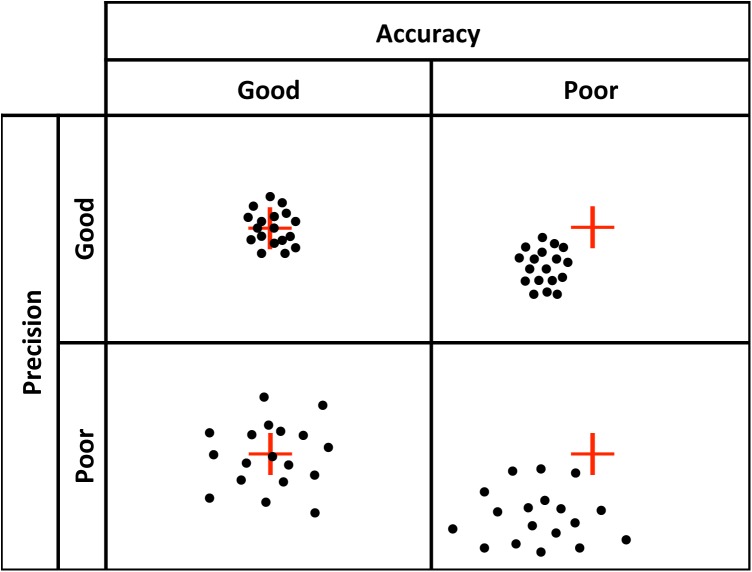
Visual representation of good vs. poor accuracy and precision.

There are at least four components to eye tracking data quality (Holmqvist et al., 2011): (1) *spatial accuracy:* the distance between the true point of gaze (POG) and the recorded POG; (2) *spatial precision*: the distance between repeated samples of POG locations when the true POG is assumed to be fairly stable, for example, when no saccade is taking place. Note that lower sample-to-sample deviation values indicate higher (better), spatial precision; (3) *temporal accuracy:* the accuracy of the reported timing of gaze events, and (4) *robustness*: the amount of data recorded relative to data lost during recording. Poor spatial accuracy (**Figure 1**) presents an obvious problem in that it creates error in determining the true location of the POG. Poor precision (**Figure 1**) is noisier data (i.e., less signal) and can influence the output of fixation and saccade classification algorithms (Holmqvist et al., 2011; Blignaut and Wium, 2014), introducing artifacts that lead to spurious shorter fixation durations (Wass et al., 2013), or longer fixation durations (Holmqvist et al., 2012; Hessels et al., 2017). Low robustness is defined by data loss, and poor temporal accuracy is the delay between the time of the eye gaze event and the reported time stamp of that event. Temporal accuracy is primarily a concern for data collected with eye trackers with lower sampling rates (e.g., 60 Hz), but can lead to concerns regarding dependent measures that rely on highly precise temporal information. Others have addressed issues of robustness (Wass et al., 2013) and temporal precision (Morgante et al., 2012). In the present study, we focus on spatial accuracy and precision, and how these metrics vary across age.

**FIGURE 2:**
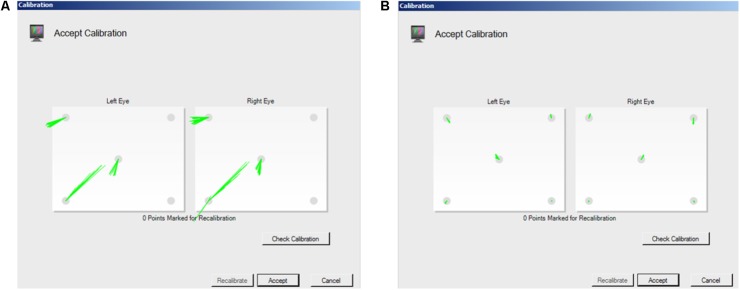
Screen shots of Tobii Studio calibration measurement. Tobii Studio’s procedure provides a qualitative visual representation of whether calibration was successful, with the accuracy of the calibration represented by the dispersion and length of the lines that mark the point. **(A)** long lines represent a relatively inaccurate calibration. **(B)** Short lines represent a relatively accurate calibration.

The accuracy and precision of a given eye tracker is directly related to how it tracks gaze. Corneal reflection is the most popular technique underlying today’s eye trackers (Holmqvist et al., 2011). Eye trackers calculate the reflection of a light source (often infra-red, which is not visible to the participant) relative to the location of the pupil, which is either illuminated, as in bright pupil tracking, or not, as in dark pupil tracking (see Holmqvist et al., 2011; Hooge et al., 2016; Nyström et al., 2016, for detailed descriptions of corneal reflection eye tracking technology). Because eye shape and size vary between individuals, a calibration is needed to calculate the corneal reflection relative to pupil location for each individual in order to compute their POG. Calibration involves placing visual targets at known locations on the screen and calculating the location of gaze as the viewer fixates those targets. The accuracy and precision of eye tracking data depend on a successful calibration (Nyström et al., 2013), and some eye trackers (e.g., SR Research Eyelink) provide a quantitative evaluation of the quality of the calibration. However, other systems simply provide a qualitative visual representation of the calibration. For example, Tobii Studio (the software that accompanies the Tobii eye tracker systems) provides a schematic representation of calibration success, marking calibration points that were successfully calibrated with error vectors (lines that indicate the difference between the POG calculated by the eye tracker and the actual location of the calibration point) and leaving calibration points blank if there was no reading (**Figure 2**). Tobii Studio also offers a qualitative calibration check, which is a dynamic visual representation of the measured location of the participant’s POG relative to nine fixed locations on the screen. The participant’s POG is displayed as a circle that moves as s/he fixates the nine targets, providing a real-time visual representation of the accuracy of the calibration. However, without a procedure in place to *quantify* the quality of the calibration, the spatial accuracy and precision of the eye tracking data for any given participant is often assumed and reported to be equal to the optimal specifications reported by the manufacturer. Although Tobii has released new software (Tobii Pro Lab) that provides measures of calibration accuracy and precision, the software is costly and researchers may have yet to upgrade. In fact, a recent survey of eye tracking researchers indicated that more than half of respondents do not even take accuracy into account when determining areas of interest (AOIs) for data analysis, potentially affecting the validity of their results and/or conclusions (Orquin et al., 2016). Thus, there is a clear need for an easy to use tool for quantifying eye tracking accuracy and precision on a participant by participant basis, and for researchers using eye tracking to understand the importance of eye tracker quality control.

Some *post hoc* calibration verification procedures have been implemented and reported by others to assess eye tracking data quality in infants and toddlers (see **Table 1**). Frank et al. (2012) used a 4-point offline calibration procedure to verify, and retroactively correct, eye tracking accuracy during a social viewing task with 3- to 30-month-old infants and toddlers using a Tobii T60XL eye tracker. Morgante et al. (2012) used a 9-point procedure to evaluate temporal and spatial accuracy of eye tracking data from the Tobii T60XL eye tracker. They found a systematic temporal delay between the true timing of a gaze event as evaluated by frame-by-frame hand-coding of video data, and the recorded timing reported by the Tobii system. In terms of spatial accuracy, they found a mean deviation of 1.27° visual angle from the true POG for a group of adults. They found no difference between the spatial accuracy measures for adults and 10-month-old infants, though after initially assessing infants and toddlers between 3 and 18 months, they excluded a number of younger infants due to data loss. They did not look at the effect of age on spatial accuracy within the infant group. Jones and Klin (2013) measured calibration accuracy for typically developing infants and infants later diagnosed with autism from 2 to 24 months. They used a video-based, 60 Hz dark pupil eye tracker (created by ISCAN), reporting the average calibration error to be on average less than 1.5 degrees of visual angle for every age group. However, this does not mean that each individual was below 1.5 degrees of error, and it is the quality of each individual’s calibration that is ultimately what matters when interpreting eye tracking data. In terms of precision, Hooge et al. (2017) computed RMS measures of the precision of their eye tracking data, which were collected from infants and adults using a Tobii TX300. They reported that the infant data were less precise than the adult data (Hooge et al., 2017). Similarly, Hessels et al. (2016) recorded data from 10-month-old infants, and adults with and without Autism Spectrum Disorders using a Tobii TX300. The infant group was more likely to have high RMS precision values, indicating poorer precision, than the adults (Hessels et al., 2016).

**Table 1:** Selected list of infant studies that provide accuracy and/or precision data.

Study	Tracker	Method
Chawarska et al. (2016)	SensoMotoric Instruments IView, 60 Hz	6-, 9-, and 12-month-old infants at high genetic risk for ASD and low risk controls.
Frank et al. (2009)	Tobii ET – 17	Excluded data from infants (3-, 6-, and 9-months-old) with average point of gaze >2° from central fixation point.
Frank et al. (2012)	Tobii T60XL	4-point offline calibration to verify and correct 3–30 month old infants and toddlers.
Hessels et al. (2015)	Tobii TX300	Explicitly tested accuracy and precision of eye tracking in 10-month-olds under various testing conditions.
Morgante et al. (2012)	Tobii T60XL	9-point procedure evaluating spatial and temporal accuracy in 3–18 month olds.
Wass et al. (2014)	TX300	Explicitly tested accuracy and precision of eye tracking in 9-, 12-, 15-month-olds.
Wass et al. (2013)	Various	Explore and discuss the effect of eye tracking data quality on fixation duration estimates in infants and adults. Discuss unique challenges associated with infant eye tracking data.

To summarize, poor spatial accuracy creates error in determining the true location of the POG, which influences interpretations about whether or not the participant is looking at a particular area of interest. This is particularly problematic when using naturalistic stimuli (e.g., scenes), where areas of interest may be small or adjacent to one another. Poor precision can influence fixation and saccade classification algorithms (Holmqvist et al., 2011; Blignaut and Wium, 2014), leading to incorrect parsing of events as fixations versus saccades. Considering these implications for infant eye tracking data quality, assessing and reporting data quality in infancy research is critical (Frank et al., 2012; Blignaut and Wium, 2014; Wass et al., 2014). However, few groups gather, let alone report, quantitative data quality information (see **Table 1** for researchers who have reported this information in studies with infants, though see van der Stigchel et al., 2017 for an excellent example of how this has been done in work with school-aged children). As mentioned above, many researchers do not take quality information, such as accuracy, into account (Orquin et al., 2016). In the current study, we sought to quantify recording accuracy/precision and examine age-related differences in groups of toddlers, school-aged children, and adults. We then examined the degree to which our results correspond with the reported manufacturer specifications. To support this effort, we designed a calibration verification procedure that can be implemented to evaluate the accuracy and precision of eye tracking data as a quality control measure for use during data collection. Although similar procedures/routines have been made available (e.g., Frank et al., 2012; Wass et al., 2013, 2014), unlike others, the procedures and processing described here are implemented in free software (Python) and our stimuli and processing script are also freely available to researchers. We also offer a user manual to facilitate easy implementation of our procedures. These tools may be particularly helpful for developmental researchers who use eye tracking as a tool to answer questions about development, but who may not be experts in the field of eye tracking *per se*. Ultimately, we believe that researchers should have a broad suite of tools to choose from to facilitate appropriate quality assessment.

Our procedure consists of a 5-point protocol for independently measuring the accuracy and precision of eye tracking data in standard lab conditions that can be used with a variety of age groups, from infants to adults. Below we describe the method and explanations for our design choices, along with the accuracy and precision of a sample of data collected with adults, school-age children, and toddlers, using a Tobii TX300 eye tracker in standard laboratory conditions (though our tool can be used with a variety of eye trackers). We discuss the implications of the results and make recommendations for modifying experimental design and data analysis to accommodate for variability in eye tracking measures. We encourage other researchers to adopt this or other similar quality assessment procedures so that the reporting of calibration quality becomes standard practice in eye tracking research.

## Material and Methods

### Participants

#### Adults

Eleven adults from the University of Minnesota (10 female, mean age = 26.6 years) were recruited to participate in this short experiment.

#### School-Age Children

Eleven children (seven female) between the ages of 8 and 11 years (mean age = 9.9 years) were recruited through the Institute of Child Development Participant Pool, a voluntary research registry operated by the Institute of Child Development at the University of Minnesota. Children were recruited as part of a larger behavioral study and participated in our quality control assessment during their 1-hour visit to the lab.

#### Toddlers

Thirty-six 18-month-old toddlers (24 female) between the ages of 18.1 and 18.97 months (mean age = 18.6 months), and thirty-six 30-month-old toddlers (17 female) between the ages of 30.0 and 30.9 months (mean age = 30.42 months) were recruited through the Infant Participant Pool. Toddlers were recruited as part of a larger study and participated in our quality control assessment at the beginning of their approximately 1.5-hour visit.

This study was approved by the Institutional Review Board at the University of Minnesota. Consent was obtained from all adult participants. Parental permission was provided for all school-aged and toddler participants. Informed assent was additionally obtained for all school-age participants. School-age children and the parents of toddlers were compensated for their participation. This study was carried out in accordance with The Code of Ethics of the World Medical Association (Declaration of Helsinki).

### Stimuli

**FIGURE 3:**
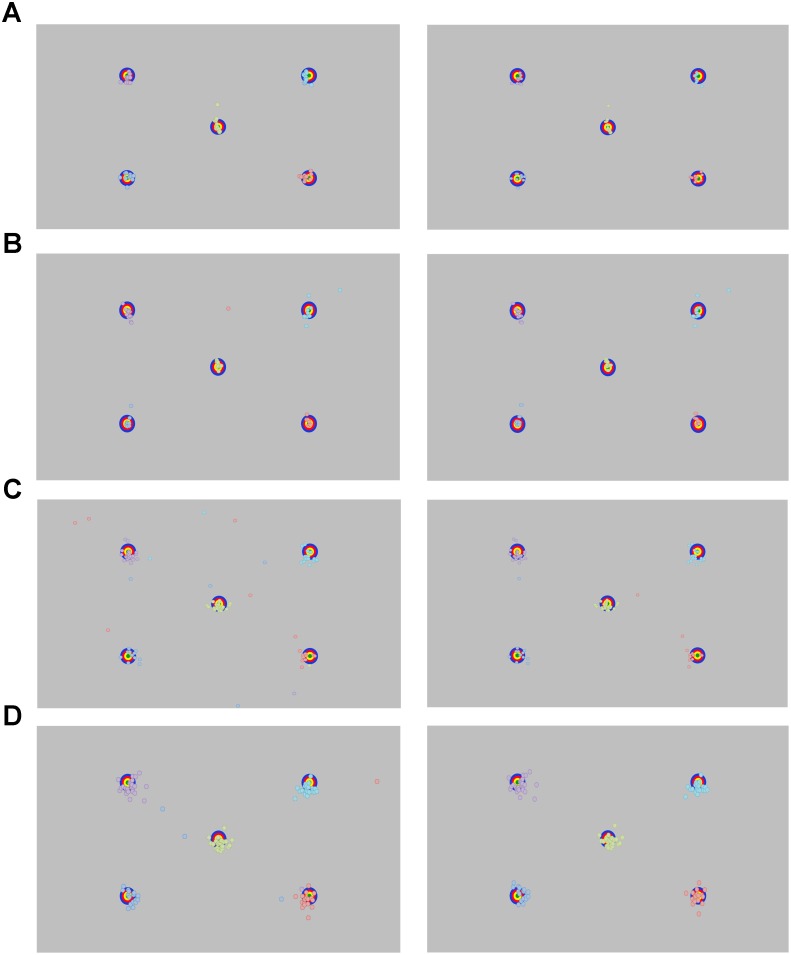
Recorded point of gaze for the longest fixation before (left panel) and after (right panel) removing outlying data for **(A)** adults, **(B)** school-aged children, and **(C)** 18-month-old and **(D)** 30-month-old toddlers.

Stimuli for adults and school-age children were five static multi-colored targets (**Figure 3**). Targets were presented on a gray background (R: 192 × G: 192 × B: 192, Hue: 160 Lum: 181) and were made up of a green circle (diameter 0.63°at a viewing distance of 60 cm), surrounded by three annuli that increased in size by 0.63°. The largest annulus (blue) was 2.52° in diameter. The stimuli were placed in the four corners of the screen at a distance of 14.1°, 8.2° (480, 270 px) from the edge of the screen, at a resolution of 1920 pixels × 1080 pixels. The fifth target was located center-screen (960, 540 px). These target locations were chosen because they sample a range of screen locations at eccentricities where stimuli are likely to appear during a typical eye tracking experiment. For toddlers, target stimuli were dynamic, with annuli disappearing and reappearing in succession, and were paired with a sound to attract their attention.

### Apparatus

Stimuli were displayed on a 27-inch ASUS wide-screen monitor set to refresh at 120 Hz. Eye gaze was tracked using a Tobii TX300 eye tracker (Tobii Technology AB, www.tobii.com). The TX300 is a desk-mounted dark pupil tracking system that has a temporal resolution of 3 ms (sampling rate 300 Hz), gaze accuracy of 0.4°-0.9°, and precision of 0.04–0.15°, depending on gaze angle and lighting, and whether noise-reduction filters are applied (see www.tobiipro.com/learn-and-support/learn/eye-tracking-essentials/ for more details about Tobii Technology manufacturer specifications).

### Procedure

#### Manufacturer Calibration

All participants were seated 60–65 cm from the screen, which is within the ideal range for recording, according to the Tobii TX300 manual. We confirmed this after testing by calculating the mean distance from screen for each group using the values measured by the TX300: Adults 64.6 cm, School-aged children 64.2 cm, Toddlers 62.0 cm. Adults and school-age children were calibrated using Tobii Studio’s 9-point automated calibration tool. This tool presents a dot that expands and contracts at nine fixed locations on the screen. Participants were instructed to look at the center of the dots. Toddlers were calibrated using Tobii Studio’s 5-point infant calibration tool, which presents audio-visual images at five fixed locations on the screen. When the experimenter judged that the toddler was looking in the direction of the stimulus for a minimum of 2 s, she advanced to the next stimulus location by key press. If a toddler directed his or her attention away from the screen, the experimenter presented a different “attention eliciting” audio-visual stimulus on the screen. The calibration stimulus was presented again when the toddler was looking in its direction. Calibration was repeated until a satisfactory calibration was obtained, based a trained researcher’s judgment of Tobii Studio’s qualitative calibration report.

#### Experimental Task

Adult and school-age participants were instructed to look at the center of the stimuli that appeared on the screen. The experiment began with a fixation cross at center-screen, followed by the five targets that appeared in pseudorandom order: random presentation except the target at center-screen was never presented first. Each target was presented for 2,000 ms. Toddler testing was the same except toddlers were seated in their caregiver’s lap and received no instructions.

### Analyses

#### Exporting Data From Eye Tracker

We exported all gaze data for each participant using Tobii Pro Studio software (Tobii Technology AB). The I-VT fixation filter was set to define the minimum fixation duration to 60 ms, with a velocity threshold of 30°/s. A fixation filter is necessary so that the processing script can identify samples that should be grouped together when calculating precision, though see Section “Discussion” where we note that fixation-classification algorithms such as the fixation filter used here can be affected by data precision (Hessels et al., 2016). The output from Tobii Pro Studio provided the participant name, media name (indicating which target was on the screen at a given time), recording timestamp, fixation index (numbering the fixations sequentially), the gaze location (average location of the left and right eye, in pixel coordinates), distance from the screen for left and right eyes, and a validity code for left and right eyes that Tobii assigns based on whether the eye was detected with certainty. Data were considered valid and were included in analysis if one or both eyes had a valid reading according to the Tobii validity criteria (i.e., eye was found with certainty). Any invalid data within a fixation were discarded.

#### Pre-processing Using In-House Python Script

The data pre-processing was conducted with a customized in-house Python script. Python is free and available to download at www.python.org and our script, along with accompanying user manual, are available for download as Supplementary Information.

A trial consists of the presentation of a single target. All participants completed all five trials. We reasoned that the longest fixation (in ms) on a given trial is most likely the fixation that was intended to land on the target. Note that we considered using the fixation that is closest to the centroid of the target, but decided that this would create a selection bias for the most accurate fixations. Indeed, others have used the longest fixation to determine spatial accuracy using similar procedures (e.g., Morgante et al., 2012). For a given participant, the script first identified the longest valid fixation for each trial. Only fixations that started after stimulus onset were considered valid. The location of the longest fixation on each trial was then calculated in pixels. Any sample that occurred outside the screen coordinates was deemed invalid and not included. Fixation location was computed by averaging valid data for the entire duration of the fixation, even if that fixation continued after stimulus offset.

Once the longest valid fixation was identified for each target location, the script computed two measures of data quality per trial: (1) Accuracy: computed as the Euclidean distance between the gaze location and the center position of the target, in degrees of visual angle, (2) Precision, calculated in two ways: the standard deviation (SD), and the root mean square (RMS), both in degrees of visual angle, and calculated in the horizontal and vertical directions (formulas from Holmqvist et al., 2011). SD is the calculated as the deviation from the mean location of all samples. RMS is calculated using the distance between successive valid gaze locations within a single fixation. In both cases lower values indicate better precision. Degrees of visual angle were calculated using the participant’s average distance from the screen for the duration of the calibration verification procedure, as computed by Tobii.

## Results

**FIGURE 4:**
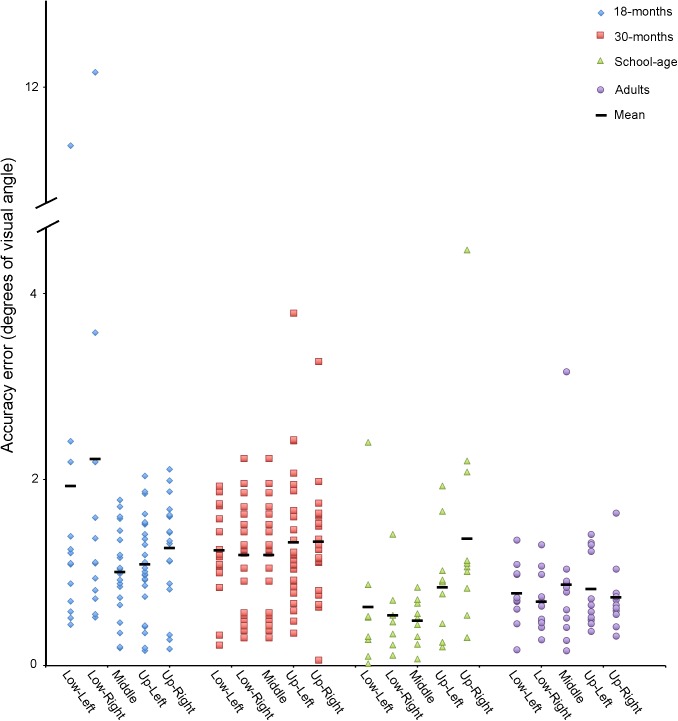
Accuracy in degrees of visual angle for adults, school-age children, and 18- and 30-month-old toddlers for each target location.

Accuracy and precision measures for each target location are reported by groups in **Figure 4** and **Table 2**.

**Table 2:** Accuracy and precision (degrees of visual angle) for adults, school-age children, and toddlers, by target location.

Target Location							
Group	Measure	Upper Left	Upper Right	Middle	Lower Left	Lower Right	Mean
Adults	Accuracy (range)	0.82 (0.37–1.41)	0.73 (0.32–1.64)	0.87 (0.16–3.16)	0.78 (0.17–1.35)	0.69 (0.28–1.30)	0.78 (0.35–1.52)
	Precision SD	0.15, 0.17	0.10, 0.19	0.09, 0.16	0.12, 0.19	0.11, 0.19	0.11, 0.18
	Precision RMS	0.11, 0.13	0.11, 0.13	0.10, 0.15	0.13, 0.20	0.12, 0.22	0.11, 0.17
School-age	Accuracy (range)	0.84 (0.20–1.93)	1.37 (0.30–4.47)	0.48 (0.07–0.84)	0.63 (0.02–2.40)	0.54 (0.11–1.41)	0.93 (0.37–2.70)
	Precision SD	0.15, 0.18	0.13, 0.20	0.12, 0.17	0.15, 0.20	0.11, 0.18	0.14, 0.19
	Precision RMS	0.15, 0.17	0.14, 0.18	0.11, 0.15	0.16, 0.21	0.11, 0.21	0.15, 0.19
Toddlers 18 months	Accuracy (range)	1.09 (0.16–2.04)	1.26 (0.18–2.11)	1.01 (0.19 –1.78)	1.93 (0.44–11.37)	2.22 (0.52–12.16)	1.31 (0.18–3.85)
Run 1	Precision SD	0.19, 0.22	0.19, 0.21	0.19, 0.19	0.19, 0.21	0.19, 0.22	0.20, 0.21
	Precision RMS	0.25, 0.24	0.23, 0.23	0.23, 0.22	0.24, 0.29	0.24, 0.26	0.25, 0.25
Run 2	Accuracy (range)	1.46 (0.34–4.26)	1.34 (0.50–2.49)	1.31 (0.40–2.04)	0.85 (0.24–2.09)	1.03 (0.22–2.21)	1.28 (0.56–2.32)
	Precision SD	0.20, 0.21	0.18, 0.20	0.18, 0.19	0.17, 0.21	0.20, 0.20	0.20, 0.21
	Precision RMS	0.24, 0.23	0.23, 0.24	0.22, 0.22	0.24, 0.27	0.22, 0.26	0.25, 0.26
Toddlers 30 months	Accuracy (range)	1.32 (0.35–3.79)	1.33 (0.06–3.27)	1.19 (0.30–2.23)	1.24 (0.22–1.93)	1.18 (0.41–2.78)	1.29 (0.67–2.33)
Run 1	Precision SD	0.18, 0.21	0.19, 0.21	0.17, 0.21	0.20, 0.22	0.18, 0.22	0.19, 0.21
	Precision RMS	0.22, 0.23	0.22, 0.25	0.22, 0.27	0.21, 0.27	0.22, 0.26	0.23, 0.27
Run 2	Accuracy (range)	1.27 (0.64–2.08)	1.54 (0.61–2.44)	1.78 (0.24–6.51)	2.06 (0.41–6.41)	2.14 (1.18–6.66)	1.77 (0.81–2.27)
	Precision SD	0.18, 0.22	0.19, 0.19	0.15, 0.19	0.16, 0.22	0.17, 0.19	0.17, 0.20
	Precision RMS	0.22, 0.23	0.23, 0.22	0.19, 0.21	0.19, 0.27	0.18, 0.24	0.21, 0.24
Tobii TX300	Accuracy						0.4–0.9^♦^
Manufacturer specifications	Precision RMS						0.04–0.15^•^

*Smaller values represent superior accuracy and precision*. ^♦^*Depends on lighting and gaze angle*^•^*Depends on whether noise-reduction filter is applied*.

### Adults

Data are plotted in **Figure 3A**. A trial is considered valid when a unique fixation (min duration 60 ms) is detected on the screen after trial onset. All adults had five out of five valid trials.

We first calculated the group’s mean Euclidean distance between POG and the target, across all targets combined. We then removed one trial that was considered an outlier, defined as falling greater than 1.5 SD above the grand mean distance from the targets. Using the remaining data, we calculated the average accuracy and precision for each of the five targets and the mean accuracy across all targets (**Table 2**). The mean accuracy across all targets was 0.78° (range: 0.35–1.52°). When looking at individual targets, accuracy ranged from 0.16° at best, to 3.16° at worst. For the precision measures, the average standard deviation across participants was 0.11°, 0.18°. RMS was 0.11°, 0.17°.

### School-Age Children

Data are plotted in **Figure 3B**. Children had between two and five out of five valid trials. The same analysis protocol that was used for the adults was used for the school-aged sample: we calculated the average Euclidean distance from POG to all targets across all participants. We removed any outliers (>1.5 SD from the mean value), which amounted to two data points total, both from the same participant. The average accuracy across all five targets was 0.93° (range: 0.37°–2.70°). When looking at individual targets, accuracy ranged from 0.02° at best, to 4.47° at worst. For our precision measures, the average standard deviation across participants was 0.14°, 0.19°. The mean RMS was 0.15°, 0.19°. See **Table 2**.

### Toddlers

#### 18-Month-Olds

Data are plotted in **Figure 3C**. We used the same assumption with the toddler gaze data as with the adult and school-age groups: that the longest fixation is the fixation that was most likely intended for the target. However, while adults and school-age children had explicit instructions to support this assumption, the toddlers did not. The toddler data were more variable, and we noted many more outliers, which may have been related, at least in part, to this lack of instructions.

We collected data from thirty-six 18-month-old toddlers. Data from eight participants were discarded for having no valid fixations. We reviewed the video recordings of these toddlers that were taken from a camera mounted on the display screen. We found that these recordings either (1) had a small number of samples because the toddler was moving excessively or not looking at the screen, or (2) the toddler was looking at the screen, but was moving his or her eyes rather than looking at the targets and therefore did not have any valid fixations (samples were classified as saccades, or “unclassified” meaning that they did not meet the criteria for a fixation or a saccade). In this case, the missing data may have been the result of poor trackability by the eye tracker. The 28 remaining participants contributed between one and five valid trials. We calculated the average Euclidean distance from POG to all targets across all participants. We removed 11 trials that were classified as outliers (>1.5 SD from the grand mean). These trials came from nine different toddlers. We then calculated the mean accuracy for each of the toddlers using the remaining trials. The average accuracy across all five targets was 1.31° (range: 0.18°–3.85°). For individual targets the accuracy was 0.16° at best, and 12.16° at worst. For our precision measures, the average *SD* across participants was 0.20°, 0.21°. The mean RMS was 0.25°, 0.25°. See **Table 2**.

After an intervening task, a subset of 21 of the 18-month-old toddlers did a second calibration verification, approximately 10 min (mean = 9 min and 34 s) after the first verification procedure. Data from 4 toddlers were removed for having no valid fixations. We analyzed the data from the remaining 17 toddlers. As with the first calibration verification, we calculated the average minimum Euclidean distance across points and removed seven trials that were >1.5 SD above the mean from 7 different toddlers. With outliers removed, we calculated the average accuracy across all five targets. The average accuracy on the second verification was 1.28° (range: 0.56°–2.32°). For individual targets the accuracy was 0.22° at best, and 4.26° at worst. For our precision measures, the average *SD* across participants was 0.20°, 0.21°. The average RMS was 0.25°, 0.26° (see **Table 2**).

A Shapiro–Wilk test of normality indicated that the data from 18-month-olds were non-normally distributed. We therefore used Wilcoxon Signed Ranks tests for related samples to compare the accuracy and precision of the first and second verification procedures to quantify change in data quality that may have occurred throughout the intervening eye tracking task. Fifteen 18-month-olds had valid data on both the first and second verification procedures. Using data from this subset of toddlers, we found that the mean accuracy did not differ significantly from the first to second calibration verification (Verification 1 = 1.29°; Verification 2 = 1.29°, Z = 0.57, *p* = 0.570), nor did the precision measures (*SD*: Verification 1 = 0.19, 0.21; Verification 2 = 0.19, 0.20. RMS: Verification 1 = 0.24, 0.24; Verification 2 = 0.24, 0.25. All *p* values >0.680).

#### 30-Month-Olds

Data are plotted in **Figure 3D**. Data from five participants were discarded for having no valid fixations. The remaining 31 participants contributed between one and five valid trials. We calculated the average Euclidean distance from POG to all targets across all participants. We removed 5 outliers total (>1.5 SD from the grand mean), which came from five different toddlers. We then calculated the mean accuracy for each of the toddlers using the remaining trials. The average accuracy across all five targets was 1.29° (range: 0.67°–2.33°). For individual targets, the accuracy was 0.06° at best, and 3.79° at worst. For our precision measures, the average SD across participants was 0.19°, 0.21°. The mean RMS was 0.23°, 0.27° (see **Table 2**).

As with the 18-month-olds, after an intervening task, a subset of 20 of the 30-month-olds did a second calibration verification, about 10 min (mean = 12 min and 29 s) after the first calibration verification procedure. Data from five toddlers were removed for having no valid fixations. We analyzed the data from the remaining 15 toddlers. We calculated the average accuracy across points and removed eight trials where the accuracy (minimum Euclidean distance) was >1.5 *SD* above the mean. These eight trials came from six different toddlers. With outliers removed, we calculated the average accuracy across all five targets. The average accuracy on the second verification was 1.77° (range: 0.81°–5.58°). For individual targets, the accuracy was 0.24° at best, and 6.66° at worst. For our precision measures, the average SD across participants was 0.17°, 0.20°. The average RMS was 0.21°, 0.24°. See **Table 2**.

A Shapiro–Wilk test of normality indicated that the data from 30-month-olds were non-normally distributed. We used Wilcoxon Signed Ranks tests for related samples to compare the accuracy and precision of the first and second verification procedures to quantify change in data quality that may have occurred throughout the intervening eye tracking task. Fourteen 30-month-olds had valid data on both the first and second verification procedures. Using data from this subset of toddlers, we found that the mean accuracy did not differ significantly from the first to second calibration verification (Verification 1 = 1.41°; Verification 2 = 1.77°, *Z* = 0.72, *p* = 0.470), nor did the precision measures (*SD*: Verification 1 = 0.18, 0.21; Verification 2 = 0.17, 0.20. RMS: Verification 1 = 0.21, 0.25; Verification 2 = 0.21, 0.24. All *p* values >0.234).

### Comparing All Groups

Shapiro–Wilk tests of normality revealed that data from school-age children and both toddler groups were non-normally distributed. We therefore compared the data from adults, school-age children, and the first run of the 18- and 30-month-old toddlers using Kruskal–Wallis non-parametric ANOVAs. We followed up any main effects with two-tail Mann–Whitney non-parametric independent samples tests, correcting for multiple comparisons (α = 0.008).

#### Accuracy

There was a significant main effect of group for Accuracy, χ^2^(3) = 17.1, *p* = .001. Mann–Whitney *post hoc* comparisons revealed that the adult calibrations were more accurate than those of the two toddler groups (vs. 18-month-olds *U* = 63.0, *p* = 0.005, vs. 30-month-olds *U* = 47.0, *p* < 0.001). The adult calibrations were no more accurate than those of the school-age children (*U* = 58.0, *p* = 0.870). The calibrations of the school-age children were more accurate than those of the 30-month-olds, *U* = 72.0, *p* = 0.005, but not the 18-month-olds, *U* = 82.0, *p* = 0.025. The accuracies of the 18- versus 30-month-old calibrations did not differ from each other, *U* = 404.0, *p* = 0.649.

#### Precision

There was a significant main effect of group for all precision measures (SDX, SDY, RMSX, and RMSY, all *p* values <0.003). Mann–Whitney *post hoc* comparisons revealed that the adult calibrations were more precise than those of the two toddler groups (all *p* values <0.005), but the adults did not differ from the school-age children (all *p* values >0.060), and the toddler groups did not differ from each other, (all *p* values >0.225). The school-age calibration data were more precise than the toddlers’ on all measures (all *p* values <0.005), except SDY (*p* values >0.009) and RMSY for school-age vs. 18-months (*p* = 0.010).

## Discussion

We designed a quality assessment protocol and examined differences in recording accuracy and precision across groups toddlers, school-aged children, and adults. We found that for adults, the mean group accuracy was within Tobii’s advertised specifications of 0.4–0.9°, and the precision measures were within or very close to the specifications of 0.04–0.15°. The data from school-aged children were on average slightly less accurate and precise than Tobii’s advertised specifications, but some children did have calibration values in the advertised range. Not surprisingly, toddler accuracy and precision was much more variable, with mean values falling beyond the advertised range for both accuracy and precision, and with some participants rejected for poor or uncertain calibration accuracy. These data illustrate the importance of quantitative measures of accuracy and precision on a participant by participant basis. Like others (e.g., Oakes, 2010; Blignaut and Wium, 2014; Wass et al., 2014; Hessels et al., 2017), we recommend that researchers who implement eye tracking studies perform independent verification of calibration accuracy and precision, and report these measures in all eye tracking studies of toddlers and children, and especially those including children with intellectual and developmental disabilities.

The accuracy and precision of our eye tracking data do not differ significantly between adults and school-aged children, but the toddler data are less accurate and less precise than adults, and for 30-month-olds less accurate than school-age children (all toddlers were less precise than school-age children on most measures). Additionally, more toddlers are rejected due to poor or uncertain calibration accuracy. These data are instructive: while mean accuracy for adult participants across all targets in our study was 0.78° (slightly less accurate than Tobii’s reported specs of 0.4°), it is important to consider the range, which was from 0.16–3.16° depending on screen location. This indicates that for some participants, the error is large enough that it could significantly alter interpretations of what the participant is fixating. For school-age children the range is even greater, 0.02–4.47° depending on location. This problem is exacerbated in toddlers, where accuracy errors as large as 12.16° for 18-month-olds and 3.79° for 30-month-olds, were observed. While it is possible that the 18-month-old with recorded accuracy error of 12.16° may not have been fixating the target (i.e., it was participant error, rather than recording error), our procedure at least flagged this participant for further inspection. Indeed, further inspection of this participant’s data indicates reasonably good accuracy for the other four target locations, with error ranging from 0.44° to 1.99°. Therefore, in this case a researcher may choose to include this participant’s eye tracking data in their results.

This case raises a key methodological consideration: because infants and toddlers cannot be instructed to look at the calibration verification stimuli, one challenge that we encountered during our analysis was determining which toddler fixations were inaccurate due to measurement error (i.e., Tobii) versus failure or unwillingness to fixate the target. Our processing script cannot distinguish between these alternatives. While we were able to exclude outliers for the purpose of summarizing accuracy and precision metrics for this study, researchers will ultimately be using our procedure, or one like it, to determine whether a toddler’s calibration is reliable enough to include that individual’s experimental data. As we did with the above case, researchers can look at the toddler’s individual data points on the five targets and determine whether the average deviation is acceptable. Some uncertainty can be resolved by reviewing video recordings of the toddler performing the calibration verification procedure: if it is clear that they are not looking at the target, that data point can be confidently discarded and others can be used to make the judgment about whether to include the toddler’s data. We recommend using a camera mounted above the display screen to record testing sessions for this reason. Researchers can also consider what magnitude of error is acceptable for their particular experiment. For example, if one is interested in fixations that land on large AOIs such as the left versus right side of the screen, a higher degree of error can be tolerated. Ideally, if there is unresolved uncertainty of the quality of a participant’s data, that participant should not be included in the data analysis.

**FIGURE 5:**
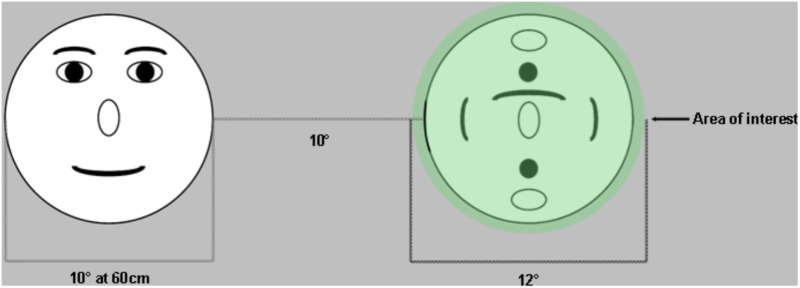
Example of stimulus design that helps accommodate accuracy error in eye tracking. Stimuli are placed far apart to ensure that there is no ambiguity with regards to which target was fixated. Areas of interest are drawn at least 1° larger than the stimuli to accommodate accuracy errors (fixations that fall within 1° of the target are assumed to be located on that target).

Although inaccurate and imprecise data are problematic, stimulus design and data analysis procedures can be modified to account for this measurement error. In terms of experimental design, stimuli can be arranged with large enough distances between areas of interest to allow for unequivocal interpretation which stimulus is being fixated (Orquin et al., 2016). Based on our data, 1.5° would be sufficient to account for this error in adults, while 2–2.5° would be more appropriate for school-age children. For toddlers, stimuli should ideally be designed with maximal distance between areas of interest (AOIs) (e.g., **Figure 5**). During data analysis, inaccuracy can be accommodated when defining AOIs (Orquin et al., 2016) (**Figure 5**). The AOIs that are mapped onto the stimuli can be drawn to exceed the stimulus by at least 1–1.5° for adults (Holmqvist et al., 2011), and >1.5° for school-age children. This means that gaze recorded on or near the AOI will be counted as landing on the AOI, accommodating accuracy error. Ideally there should be space between adjacent AOIs, but this is not always possible (e.g., when examining looks at eyes within a face when both the eyes and the remainder of the face are of interest). In contrast to accuracy, precision error essentially amounts to noisier signal and particularly affects fixation and saccade algorithms (Holmqvist et al., 2011). Some algorithms have been specifically designed to accommodate low quality data (Hessels et al., 2017; van Renswoude et al., 2017). Large AOIs can also be helpful here because they are more accommodating to noisier data (Hessels et al., 2016).

Following from these experiment results, we also recommend that researchers perform analyses to determine whether their dependent measure(s) is orthogonal to their accuracy and precision measures. For example, because poor precision can result in the artifact of shorter fixation durations (Wass et al., 2013), if the primary dependent measure is fixation duration, it is critical to evaluate whether precision is associated with study outcomes. Similarly, given that accuracy can vary by screen location, poor accuracy could differentially affect dwell time and fixation count measures for different AOIs, especially if those AOIs appear in consistent locations relative to each other on the screen (e.g., for face stimuli, eyes are always located higher on the screen than the mouth).

Others have reported that eye tracking data may become less accurate over time, a difficulty known as “drift” (Frank et al., 2012; Morgante et al., 2012). Hessels et al. (2015) found that data from 10-month-old infants became less precise and less robust over time. We did not find significant changes in accuracy or precision in our toddler data, but again, a number of toddlers were excluded from the analysis for having no valid data on either the first or second verification procedure. Ultimately, depending on the duration of the experimental task, it could be useful to implement a calibration verification procedure before, after, and perhaps throughout the task to measure drift (Shultz et al., 2011), and also the reliability of the measurements.

In some cases, calibration will be too inaccurate for the data to be included. While it may be satisfying to the reader to receive an explicit recommendation inclusion/exclusion criteria that can be applied to all eye tracking data, these criteria will ultimately depend on the idiosyncrasies of the experimental protocol (e.g., stimulus design, participant characteristics, discussed above). We identified individual trials within our verification procedure where accuracy error was greater than 1.5 SD above the mean. A more sophisticated solution would be to use the calibration verification procedure to identify the direction of the error and to correct gaze data accordingly. However, our data suggest that there is limited consistency with regard to the direction of accuracy error, with some error in the positive direction and other in the negative direction, within the same participant. Thus, it may be safest to discard the data. With adults, few participants will be excluded due to poor calibration, but we found that about 20% of toddlers could be excluded based on poor calibration alone. This does not include toddlers who would be excluded for not performing the experimental task itself. For toddlers, we recommend investigating the data from individuals with mean error greater than 1.5 SD above the mean or more by watching the video recordings to determine whether the source of error is indeed measurement error, or if instead the toddler was not looking at the targets.

To summarize, these results add to a growing literature calling attention to improved characterization of eye tracking data quality (e.g. Oakes, 2010; Blignaut and Wium, 2014; Wass et al., 2014; Hessels et al., 2017). We have summarized why data quality matters, and how it can impact study results. We have also discussed options for accommodating poor accuracy and precision during study design and data analysis, and have provided sample data from four different groups to illustrate how data quality varies depending on participant age. Our data also illustrate how data quality compares to manufacturer specifications, which are often reported instead of values computed from the data that are being presented. Our stimuli, data processing scripts, sample data, and user manual are available for download online as Supplementary Material. Although we implemented our method with a Tobii TX300 eye tracker, our protocol may be used with other eye trackers, providing that they can output the critical information required for the script listed in our user manual. While Tobii Inc. has released a new tool (Tobii Pro Lab) that provides some quantitative measures of calibration accuracy and precision, our tool provides an external measure of data quality and is available to download for free. We recommend that this (or a similar) quality assessment procedure become standard protocol for the preprocessing of eye tracking data, particularly when collecting data from toddlers and children with intellectual and developmental disabilities. Rather than reporting the eye tracker’s advertised specifications, we encourage researchers to report the mean accuracy and precision from the data and to take these measures into account when analyzing and reporting data. This could have a significant impact on the quality of the data published and on the conclusions drawn from eye tracking studies.

## Author Contributions

KD and JE contributed to the design and implementation of the study. KD, KH, and ET contributed to the collection and analysis of the data. KD and MM contributed to the design and implementation of the analysis tool. KD, KH, MM, ET, and JE contributed to the preparation of the manuscript.

## Conflict of Interest Statement

The authors declare that the research was conducted in the absence of any commercial or financial relationships that could be construed as a potential conflict of interest.
