# Supplementary material for: An Examination of Recording Accuracy and Precision From Eye Tracking Data From Toddlerhood to Adulthood
Source: Front Psychol. 2018 May 23;9:803. doi: 10.3389/fpsyg.2018.00803 (PMC5974590; doi:10.3389/fpsyg.2018.00803)
Supplement: Supplementary file 3 [file Image_1.PDF]

User Notes for Calibration Verification Script

Script was written by Marie D. Manner, to accompany "An examination of recording accuracy and precision from eye tracking data from toddlerhood to adulthood", by Dalrymple, Manner, Harmelink, Teska, & Elison.

Background:
Calibration.py was written to calculate the accuracy and precision of eye tracking data during the calibration verification task designed by Dalrymple et al. Accuracy is defined as the Euclidean distance between the location of gaze and the target. Precision is defined as the standard deviation (SD) or root mean square (RMS) of samples that are used to make a fixation. This script was written to accompany eye movement data output through Tobii Studio, but can be used with output from other eye trackers as long as the data is organized according the instructions below (see Implementation).

Requirements:
Download Python from www.python.org. Requires Version 3.0 or above. Version 3.3 recommended.

Implementation:

1. Collect eye movement data from participants using the Calibration Verification stimuli (available for download).
2. Set a fixation filter to define fixations. We recommend a minimum duration of 60ms with a velocity threshold of 30°/s.
3. Output data columns listed in Table 1 in the order listed. Header names should appear exactly as shown here, case sensitive (See Figure 1).
4. Save as .csv file, one recording/participant per file.
5. Put all .csv files in one folder and save on the desktop.
6. Open calibration.py by double clicking on the file.
7. Modify the script if needed: Rename the stimuli, adjust calibration stimulus locations (Lines 61-65)
8. Select script window, Run→ Run Module (F5 on Mac).
9. When prompted, select the folder that contains the .csv data files.
10. When prompted, input screen size (height and width) in mm and screen resolution (height and width) in pixels.
11. Output for all .csv files will appear on the desktop in the format "FolderName_output.csv". This will be accompanied by a text file "FolderName_summary.txt" that lists the files that were processed versus rejected due to insufficient information (e.g. if there are no valid fixations for any stimuli).

Table 1. Input

Column	Column Header (row 1)	Description
A	ParticipantName	Participant ID
B	RecordingDate	Date of recording (optional)
C	FixationFilter	Type of fixation filter used (optional)
D	MediaName	Name of stimuli. These should match the calibration verification stimuli used (e.g. TopRight.jpg, or TopRight.avi) Names must be exactly as they appear in the Python script, case sensitive.
E	RecordingTimestamp	Recording time, in ms.
F	FixationIndex	Fixations numbered sequentially, 1, 2, 3…
G	GazeEventType	Fixation, saccade, or unclassified (optional)
H	GazeEventDuration	Duration of the event, in ms.
I	GazePointX (ADCSpx)	X coordinate for gaze location for average of two eyes (can use left or right eye if preferred).
J	GazePointY (ADCSpx)	Y coordinate for gaze location for average of two eyes (can use left or right eye if preferred).
K	DistanceLeft	Distance between the left eye and the eye tracker, in mm.
L	DistanceRight	Distance between the right eye and the eye tracker, in mm.
M	ValidityLeft	Tobii validity code for reliability of gaze data from left eye. 0=valid , 4=invalid.
N	ValidityRight	Tobii validity code for reliability of gaze data from right eye. 0=valid, 4=invalid.

Table 2. Output explained. See Figure 2 for sample output.

Column	Description
Stimulus………………………………………………..	Stimulus name (represents the location of the target on the screen).
Min Euclidean dist. (degrees) ………………..	The minimum Euclidean distance between the longest fixation and the centroid of the stimulus in degrees of visual angle.
Coordinates X …………………………..………..	X coordinate for the location of the longest fixation (pixels).
Coordinates Y …………………………..………..	Y coordinate for the location of the longest fixation (pixels).
Duration (ms)………………………………………..	Duration of the longest fixation in ms.
Precision SD X ……………………………………	Standard deviation of the samples making up the longest fixation in the X direction (degrees of visual angle).
Precision SD Y ……………………………………	Standard deviation of the samples making up the longest fixation in the Y direction (degrees of visual angle).
Precision RMS X ………………………………...	Root Mean Square of the samples making up the longest fixation in the X direction (degrees of visual angle).
Precision RMS Y ………………………………...	Root Mean Square of the samples making up the longest fixation in the Y direction (degrees of visual angle).

Technical information:

- Script only uses data with at least one valid eye.
- Fixations must begin during the stimulus presentation to be counted for that stimulus. If the fixation begins prior to stimulus onset, it will not be counted.
- Our procedure used targets centered at specific pixel locations on the screen; if you have a different screen size or different location of stimuli, you can update that information on lines 60 - 65 of the script. If, for example, your screen is half the size of our 1920 x 1080 monitor, you should change your stimuli locations from the current settings:

```
locations = {"Fix.jpg": [960.0, 540.0],
"TopLeft.jpg": [480.0, 270.0],
"TopRight.jpg": [1440.0, 270.0],
"Middle.jpg": [960.0, 540.0],
"BottomLeft.jpg": [480.0,810.0],
"BottomRight.jpg": [1440.0, 810.0]}
```

to your own locations and names, which could be, for example:

```
locations = {"SampleFix.jpg": [480.0, 270.0],
"TopL.png": [240.0, 135.0],
"TopR.png": [720.0, 135.0],
"Mid.png": [480.0, 270.0],
"BottomL.png": [240.0,405.0],
"BottomR.png": [720.0, 405.0]}
```

Glossary:

- **Accuracy:** the Euclidean distance between the location of gaze and the target.
- **Precision:** the standard deviation (SD) or root mean square (RMS) of samples that are used to make a fixation.
- **Standard Deviation (SD):** calculated using deviation from the mean location of all samples. A higher SD indicates lower precision.
- **Root Mean Square (RMS):** RMS of Euclidean distance between samples when point of gaze remains constant. A higher RMS indicates lower precision.

Figure 1. Sample input.

	A	B	C	D	E	F	G	H	I	J	K	L	M	N
1	ParticipantName	RecordingDate	FixationFilter	MediaName	RecordingTimestamp	FixationIndex	GazeEventType	GazeEventDuration	GazePointX (ADCSpx)	GazePointY (ADCSpx)	DistanceLeft	DistanceRight	ValidityLeft	ValidityRight
2	Participant1	4/27/17	I-VT filter	BottomLeft.jpg	3969	9	Fixation	207	507	761	649.13	649.13	0	0
3	Participant1	4/27/17	I-VT filter	BottomLeft.jpg	3973	9	Fixation	207	505	768	649.12	649.12	0	0
4	Participant1	4/27/17	I-VT filter	BottomLeft.jpg	3976	9	Fixation	207	510	759	649.12	649.12	0	0
5	Participant1	4/27/17	I-VT filter	BottomLeft.jpg	3979	9	Fixation	207	509	764	649.13	649.13	0	0
6	Participant1	4/27/17	I-VT filter	BottomLeft.jpg	3983	9	Fixation	207	505	763	649.09	649.09	0	0
7	Participant1	4/27/17	I-VT filter	BottomLeft.jpg	3986	9	Fixation	207	508	764	649.09	649.09	0	0
8	Participant1	4/27/17	I-VT filter	BottomLeft.jpg	3989	9	Fixation	207	508	756	649.07	649.07	0	0
9	Participant1	4/27/17	I-VT filter	BottomLeft.jpg	3993	9	Fixation	207	504	758	649.06	649.06	0	0
10	Participant1	4/27/17	I-VT filter	BottomLeft.jpg	3996	9	Fixation	207	504	765	649.05	649.05	0	0
11	Participant1	4/27/17	I-VT filter	BottomLeft.jpg	3999	9	Fixation	207	506	771	649.05	649.05	0	0
12	Participant1	4/27/17	I-VT filter	BottomLeft.jpg	4003	9	Fixation	207	506	756	649.04	649.04	0	0
13	Participant1	4/27/17	I-VT filter	BottomLeft.jpg	4006	9	Fixation	207	508	754	649.03	649.03	0	0
14	Participant1	4/27/17	I-VT filter	BottomLeft.jpg	4009	9	Fixation	207	507	762	649.02	649.02	0	0
15	Participant1	4/27/17	I-VT filter	BottomLeft.jpg	4013	9	Fixation	207	504	763	649.02	649.02	0	0
16	Participant1	4/27/17	I-VT filter	BottomLeft.jpg	4016	9	Fixation	207	501	761	649	649	0	0
17	Participant1	4/27/17	I-VT filter	BottomLeft.jpg	4019	9	Fixation	207	507	757	649	649	0	0
18	Participant1	4/27/17	I-VT filter	BottomLeft.jpg	4023	9	Fixation	207	500	761	649	649	0	0
19	Participant1	4/27/17	I-VT filter	BottomLeft.jpg	4026	9	Fixation	207	503	767	648.99	648.99	0	0
20	Participant1	4/27/17	I-VT filter	BottomLeft.jpg	4029	9	Fixation	207	503	762	649	649	0	0
21	Participant1	4/27/17	I-VT filter	BottomLeft.jpg	4033	9	Fixation	207	503	767	648.98	648.98	0	0
22	Participant1	4/27/17	I-VT filter	BottomLeft.jpg	4036	9	Fixation	207	505	769	648.98	648.98	0	0
23	Participant1	4/27/17	I-VT filter	BottomLeft.jpg	4039	9	Fixation	207	504	769	648.94	648.94	0	0
24	Participant1	4/27/17	I-VT filter	BottomLeft.jpg	4043	9	Fixation	207	507	766	648.96	648.96	0	0
25	Participant1	4/27/17	I-VT filter	BottomLeft.jpg	4046	9	Fixation	207	502	758	648.97	648.97	0	0
26	Participant1	4/27/17	I-VT filter	BottomLeft.jpg	4049	9	Fixation	207	504	764	648.95	648.95	0	0
27	Participant1	4/27/17	I-VT filter	BottomLeft.jpg	4053	9	Fixation	207	507	763	648.95	648.95	0	0
28	Participant1	4/27/17	I-VT filter	BottomLeft.jpg	4056	9	Fixation	207	506	763	648.93	648.93	0	0
29	Participant1	4/27/17	I-VT filter	BottomLeft.jpg	4059	9	Fixation	207	503	761	648.91	648.91	0	0
30	Participant1	4/27/17	I-VT filter	BottomLeft.jpg	4063	9	Fixation	207	504	758	648.91	648.91	0	0
31	Participant1	4/27/17	I-VT filter	BottomLeft.jpg	4066	9	Fixation	207	502	769	648.9	648.9	0	0
32	Participant1	4/27/17	I-VT filter	BottomLeft.jpg	4069	9	Fixation	207	504	766	648.87	648.87	0	0

Figure 2. Sample output.

	A	B	C	D	E	F	G	H	I	J	K
1	Participant1										
2	Stimulus	Min Euclidea	Coordinates	Coordinates	Duration (ms	Precision SD	Precision SD	Precision RM	Precision RMS Y		
3	BottomLeft.j	0.69	474	786.47	323	0.13	0.14	0.12	0.15		
4	BottomRight	0.69	1456.28	791.83	1893	0.12	0.3	0.08	0.18		
5	Middle.jpg	3.16	961.58	428.36	1746	0.09	0.15	0.07	0.11		
6	TopLeft.jpg	1.41	450.24	310.05	1049	0.12	0.12	0.09	0.11		
7	TopRight.jpg	1.64	1457.21	325.12	720	0.07	0.23	0.08	0.12		
8	Averages:	1.52	959.86	528.37	1146.2	0.1	0.19	0.09	0.13		
9	Number valic	5 / 5 points									
10	Participant2										
11	Stimulus	Min Euclidea	Coordinates	Coordinates	Duration (ms	Precision SD	Precision SD	Precision RM	Precision RMS Y		
12	BottomLeft.j	3.16	493.64	700.82	859	0.11	0.15	0.13	0.13		
13	BottomRight	2.83	1459.31	713.21	1300	0.17	0.3	0.09	0.15		
14	Middle.jpg	0.09	956.82	539.67	1539	0.06	0.13	0.07	0.1		
15	TopLeft.jpg	3.42	512.71	384.4	1616	0.08	0.12	0.06	0.07		
16	TopRight.jpg	0.84	1428.35	296.79	1390	0.08	0.18	0.07	0.08		
17	Averages:	2.07	970.16	526.98	1340.8	0.1	0.18	0.08	0.11		
18	Number valic	5 / 5 points									
19	Participant3										
20	Stimulus	Min Euclidea	Coordinates	Coordinates	Duration (ms	Precision SD	Precision SD	Precision RM	Precision RMS Y		
21	BottomLeft.j	3.46	473.18	692.59	1720	0.14	0.16	0.08	0.09		
22	BottomRight	2.46	1457.35	728.09	1803	0.1	0.27	0.07	0.12		
23	Middle.jpg	1.06	966.82	504.64	2220	0.06	0.16	0.06	0.07		
24	TopLeft.jpg	3.36	485.99	384.06	566	0.08	0.08	0.06	0.07		
25	TopRight.jpg	1.68	1423.81	324.93	1917	0.07	0.19	0.06	0.07		
26	Averages:	2.4	961.43	526.86	1645.2	0.09	0.17	0.07	0.08		
27	Number valic	5 / 5 points									
